# Genetic Characteristics Associated with Probiotic Functions in Four Indonesian Skin Microbiome-Derived Bacterial Strains

**DOI:** 10.3390/microorganisms14010248

**Published:** 2026-01-21

**Authors:** Ahmad Husein Alkaff, Amarila Malik, Patricia Arabela Situmeang, Nicholas C. K. Heng

**Affiliations:** 1 Department of Chemistry, Faculty of Mathematics and Natural Sciences, Universitas Indonesia, Depok 16424, Indonesia; ahmad.husein@sci.ui.ac.id; 2 Division of Pharmaceutical Microbiology and Biotechnology, Faculty of Pharmacy, Universitas Indonesia, Depok 16424, Indonesia; patricia.arabela@ui.ac.id; 3 Sir John Walsh Research Institute, Faculty of Dentistry, University of Otago, Dunedin 9054, New Zealand

**Keywords:** skin microbiome-derived bacteria, bacterial whole-genome sequencing, *Bacillus subtilis*, *Micrococcus luteus*, *Staphylococcus hominis*, *Staphylococcus warneri*, bioinformatics, probiotic functions, bacteriocins

## Abstract

The human skin microbiome has gained considerable attention as a resource for the development of innovative probiotics for cosmetic purposes or promoting skin health. However, the evaluation of new probiotic strains to ensure their “generally recognized as safe” (GRAS) status remains challenging. Here, we have subjected the annotated draft genome sequences of four human skin-derived bacterial strains, namely *Bacillus subtilis* MBF10-19J, *Micrococcus luteus* MBF05-19J, *Staphylococcus hominis* MBF12-19J, and *Staphylococcus warneri* MBF02-19J, to bioinformatic analyses to detect the genes associated with important probiotic traits, as well as undesirable characteristics such as antibiotic resistance, virulence factors, and toxic metabolites. Each bacterium harbors at least one type of adhesin-encoding gene, while only *S. hominis* MBF12-19J and *S. warneri* MBF02-19J contain the putative genes encoding enzymes for metabolism improvement. In vitro assays, including antibiotic susceptibility and antimicrobial activity testing, revealed strain-specific safety characteristics that complement the genomic findings. With regard to antibiotic resistance determinants, *S. hominis* MBF12-19J showed the most favorable profile, *S. warneri* MBF02-19J and *M. luteus* MBF05-19J appeared suitable when used with appropriate caution, and *B. subtilis* MBF10-19J exhibited amoxicillin resistance, i.e., warrants careful evaluation. Further in vivo validation is needed to determine whether these strains do indeed comply with GRAS evaluation frameworks.

## 1. Introduction

The investigation of the diversity and functions of the human skin microbiome has gained considerable attention in dermatological research. This has led to a heightened awareness of the microorganisms residing in symbiosis with humans, occupying diverse skin niches. These microorganisms play a pivotal role in safeguarding our skin by actively countering pathogens, controlling inflammation, and modulating adaptive immune pathways [1]. Recent studies indicate that the topical application of skin probiotics not only alleviates conditions like acne and atopic dermatitis but also yields cosmetic benefits for healthy skin, including moisturization and mitigation of aging effects. Consequently, the utilization of skin-dwelling bacteria and their derived metabolites emerges as a promising avenue for developing innovative microbiome-based components for cosmetics or promoting skin health [2,3].

Previously, several beneficial commensal bacterial species were isolated from Indonesian young adults’ facial skin microbiomes, including *Bacillus subtilis* MBF10-19J, *Micrococcus luteus* MBF05-19J, *Staphylococcus hominis* MBF12-19J, and *Staphylococcus warneri* MBF02-19J [4]. Subsequent studies revealed that a bacterial cocktail containing these strains not only demonstrated potent activity in terms of inhibiting the growth of *Cutibacterium* (formerly *Propionibacterium*) *acnes*, along with mild-to-low antiradical-scavenging activity, but also caused no adverse reactions in a skin sensitivity test, demonstrating its safety for use on the skin [5]. Furthermore, the ability of these species to coexist in a bacterial cocktail has also been demonstrated [6].

Despite the promising potential of microbiome-derived probiotic strains for skin-care use, their safety evaluation remains challenging. While most organisms commonly used as probiotics (e.g., gut bacteria) have been granted “generally regarded as safe” (GRAS) status, the ongoing emergence of new strains, partly driven by environmental factors, necessitates the comprehensive assessment of each novel strain for their probiotic, and more importantly, safety characteristics. These assessments encompass taxonomic identification, safety analysis, pathogenicity assessment (including adverse effects), antibiotic resistance, and whole-genome sequencing [7,8,9]. Assigning GRAS status to new probiotic-candidate strains is inappropriate because it assumes that a new strain shares the same documented safety history as a pre-existing probiotic strain of the same species [10]. When addressing the challenging safety assessment process and rising concerns about the potential risks of probiotic products on human health, whole-genome sequencing (WGS) technology and genomic analysis have proven to be robust tools for predicting genetic stability, antibiotic resistance, virulence factors, and toxin production [11,12,13].

Here, we report on the bioinformatic analyses of the genome sequences of four potential skin microbiome-derived probiotic candidates, namely *B. subtilis* MBF10-19J, *M. luteus* MBF05-19J, *S. hominis* MBF12-19J, and *S. warneri* MBF02-19J. Among the genetic determinants we focused on were transferable antimicrobial resistance (AMR) genes, potential virulence factor (VF)-encoding genes, and genes possibly related to toxic metabolite production. In addition, we also checked for the presence of potential probiotic-related genes, bacteriophage-related (e.g., prophage and CRISPR/Cas) loci, and mobile genetic elements (insertion sequences) within the genome sequences of these isolated strains. Using in vitro assays, we also tested whether the putative AMR and bacteriocin-encoding genes were functional. Our findings provide supportive evidence for these four candidate strains as human skin probiotics.

## 2. Materials and Methods

### 2.1. Whole-Genome Sequencing of Microbiome-Derived Bacterial Strains

*B. subtilis* MBF10-19J, *M. luteus* MBF05-19J, *S. hominis* MBF12-19J, and *S. warneri* MBF02-19J all originated from facial skin microbiome samples collected from a young healthy Indonesian volunteer, as detailed previously [4]. These strains were maintained at 37 °C on either nutrient agar (*B. subtilis*), blood agar (*M. luteus*), or tryptic soy agar (*S. hominis* and *S. warneri*). Genomic DNA was extracted from 10 mL overnight (18–22 h) cultures of each bacterial strain using the Presto™ Mini gDNA Bacteria (Geneaid, New Taipei City, Taiwan) Kit, following the manufacturer’s protocol. One nanogram of each genomic DNA sample was subjected to whole-genome shotgun sequencing on an Oxford Nanopore GridION system (PT Genetika Science, Jakarta, Indonesia). De novo assembly of each genome was carried out using Flye v2.8.1 [14]. Each genome sequence was annotated using the NCBI Prokaryotic Genome Annotation Pipeline (PGAP) v4.6 [15] and the Rapid Annotations using Subsystems Technology (RAST) v2 server [16].

### 2.2. Genetic Assessment Procedures

#### 2.2.1. Detection of Putative Beneficial Probiotic-Related Genes

The coding sequences associated with pivotal probiotic genes, including the production of lactic acid, adhesion to epithelial tissue, and enhancement of host metabolism, were elucidated through the functional annotation of coding genes.

#### 2.2.2. Identification of Antibiotic Resistance and Bacteriocin Genes, and Virulence Determinants

Identification of antibiotic resistance genes was performed by utilizing the Comprehensive Antibiotic Resistance Database (CARD) (http://arpcard.mcmaster.ca/), limiting the hits to “perfect” and “strict” criteria only [17], and ResFinder 4.4.2 (https://genepi.food.dtu.dk/resfinder, accessed on 15 January 2026), covering 90% of %ID threshold and minimum length of 60% [18]. Bacteriocin-related genes were identified using BAGEL4 (http://bagel4.molgenrug.nl) utilizing only the Class I (known bacteriocins and/or ribosomally synthesized and post-translationally modified peptides [RiPPs]) and Class II (putative or novel bacteriocins and/or RiPPs) parameters [19]. Putative genes encoding virulence factors were identified using the Virulence Factor Database (VFDB) (http://www.mgc.ac.cn/VFs/, accessed on 15 January 2026) with the default blastn algorithm [20].

#### 2.2.3. Prediction of Genes Related to Production of Toxic Metabolites

The prediction of genes potentially linked to toxic metabolites in the four bacterial genome sequences involved manual searching of each annotated genome sequence for open reading frames (ORFs) encoding enzymes directly related to the production of biogenic amines, e.g., tyrosine decarboxylase, histidine decarboxylase, ornithine decarboxylase, L-lysine decarboxylase, agmatine dehydrolase, and agmatine deiminase, as well as nitrogenous compounds (e.g., nitroreductase and nitrate reductase). In addition, manual searching for the presence of genes involved in toxin production, such as cytotoxin K and hemolysin, as well as those associated with lipopeptide synthesis, including fengycin, lychenysin, and surfactins, was also carried out for each genome [11].

#### 2.2.4. Assessment of Genome Stability

To assess the genome stability of each whole-genome sequence, we also looked for the presence of Clustered Regularly Interspaced Short Palindromic Repeats (CRISPR) and CRISPR-associated gene (Cas) sequences, prophage sequences, and bacterial insertion sequences (IS). We also looked at the coding sequences for CRISPR-Cas using the CRISPRCasMeta tools (https://crisprcas.i2bc.paris-saclay.fr/CrisprCasMeta/Index, accessed on 15 January 2026) by employing default parameters for detecting sequences at “high homology” level [21]. Putative prophage sequences were identified using Phage Search Tool Enhanced Release (PHASTER) (https://phaster.ca/) utilizing default parameters to detect intact, questionable, and incomplete prophage regions [22]. IS elements were detected using ISfinder (www-is.biotoul.fr) using default parameters, with the IS family exhibiting *E* values of <1.00 *×* 10^−10^ being of further interest [23].

### 2.3. In Vitro Assays

#### 2.3.1. Antibiotic Sensitivity Assays

Antibiotic sensitivities of each bacterial strain were examined using the disk diffusion method and minimum inhibitory concentration (MIC) microbroth dilution technique, according to standard Clinical and Laboratory Standards Institute (CLSI) protocols. Mueller–Hinton agar (MHA) (Oxoid^TM^ Thermo Fisher Scientific, Basingstoke, UK) was used as the test medium. For the disk diffusion method, overnight broth cultures of each strain were suspended to the 0.5 McFarland turbidity standard. The standard antibiotic disks that were used for these assays were vancomycin 30 μg, gentamycin 10 μg, chloramphenicol 30 μg, erythromycin 15 μg, amoxicillin 25 μg, and ciprofloxacin 5 μg (all supplied by Oxoid^TM^ Thermo Fisher Scientific, Basingstoke, UK). These disks were applied on inoculated agar plates. Plates were incubated at 37 °C for 24 h, and zones were measured and interpreted for susceptibility [24,25].

Minimum inhibitory concentrations were determined by broth microdilution using Mueller–Hinton broth (MHB) (HiMedia, Mumbai, India) as the test medium. This assay was conducted in 96-well microtiter plates (Biologix, Camarillo, CA, USA) (total volume 200 μL) for all four probiotic strains with regard to vancomycin resistance, and only *B. subtilis* for amoxicillin resistance. Vancomycin (Hospira Inc., Lake Forest, IL, USA) and amoxicillin (Sigma-Aldrich, St Louis, MO, USA) were prepared in appropriate diluents as recommended by the manufacturer; vancomycin was serially diluted from 32 μg/mL to 1 μg/mL, and amoxicillin from 100 μg/mL to 0.78 μg/mL. Probiotic suspensions containing 10^6^ CFU/mL were prepared and we inoculated 100 μL into MHB to a final concentration of 10^5^ CFU/mL. The same procedure was conducted for *B. subtilis* antibiotic resistance. Amoxicillin assays were incubated at 37 °C for 16–20 h, while vancomycin plates were incubated for 24 h. Well turbidity was measured using a microplate reader (GloMax® Discover Microplate Reader, Promega Corp., Madison, WI, USA) with 600 nm and interpreted for susceptibility [24,25]. Each test was carried out in triplicate.

#### 2.3.2. Antimicrobial Assays

Antimicrobial potential was determined with a deferred antagonism cross-streak method. Mueller–Hinton agar (Oxoid^TM^ Thermo Fisher Scientific, Basingstoke, UK) was prepared, each probiotic strain was inoculated as a 1-cm streak in the center of each plate, and incubated at 37 °C for 48 h. Indicator bacteria included a selection of Gram-positive species (*Streptococcus mutans*, *Staphylococcus aureus*, and *B. subtilis*), and Gram-negative bacteria (*Pseudomonas aeruginosa*, *Salmonella typhimurium*, and *Escherichia coli*). Indicator bacteria were adjusted to 10^9^ CFU/mL and streaked perpendicularly to the center line without touching the center streak and incubated at 37 °C for 24 h. Antimicrobial activity was considered positive if there was growth inhibition [26,27].

## 3. Results

### 3.1. Genome Sequence Characteristics of B. subtilis, M. luteus, S. hominis, and S. warneri

Genomic sequence quality was assessed after being subjected to whole-genome shotgun sequencing on an Oxford Nanopore GridION system (Supplementary Table S1). The assembled whole-genome sequences of *B. subtilis* MBF10-19J (4,124,541 bp long in two contigs), *M. luteus* MBF05-19J (2,588,352 bp in two contigs), *S. hominis* MBF12-19J (2,295,496 bp in two contigs), and *S. warneri* MBF02-19J (2,521,958 bp in twelve contigs) have been deposited in GenBank under accession numbers JAGMTL000000000, JAGMUB000000000, JAGMUC000000000, and JAGMUP000000000, respectively. All assembled genomes showed high sequencing coverage, ranging from 230.0- to 275.0-fold, and demonstrated good overall quality. Their completeness values ranged from 89.28 to 99.65 percent, while contamination levels remained low, between 0.06 and 5.12 percent. Collectively, these metrics indicated that all genomes were suitable for the downstream bioinformatic analyses conducted in this study (Table 1).

### 3.2. Probiotic Potential

#### 3.2.1. L-Lactate Dehydrogenase and D-Lactate Dehydrogenase

The annotated whole-genome sequences from each strain were examined for the coding sequences associated with beneficial probiotic properties such as lactate dehydrogenase, which is responsible for lactic acid production (Table 2). *B. subtilis* MBF10-19J, *M. luteus* MBF05-19J, *S. hominis* MBF12-19J, and *S. warneri* MBF02-19J each have one coding sequence (CDS) for L-lactate dehydrogenase (EC 1.1.1.27). In addition, both *S. hominis* MBF12-19J and *S. warneri* MBF02-19J each have two CDSs encoding D-lactate dehydrogenase (EC 1.1.1.28) (Table 2).

#### 3.2.2. Cell Signaling and Adhesion Encoding Genes

Several coding sequences identified in the Indonesian skin isolates could be involved in adhesion to the surrounding epithelial tissue (Table 3). CDS encoding tyrosine-protein kinase was found in *B. subtilis* MBF10-19J, *M. luteus* MBF05-19J, and *S. hominis* MBF12-19J. The presence of tyrosine-protein kinases is vital in cell signaling and cell adhesion [29]. In addition, CDSs encoding a class A sortase was found in *S. hominis* MBF12-19J and *S. warneri* MBF02-19J, while a class F sortase was only found in *M. luteus* MBF05-19J. Class A sortases are conserved transpeptidase enzymes that catalyze covalent bonds between many surface proteins to the cell wall peptidoglycan [30].

Among the four bacterial strains analyzed, only *S. hominis* MBF12-19J and *S. warneri* MBF02-19J contain CDS encoding enzymes in connection with metabolism improvement, such as poly(glycerol-phosphate) alpha-glucosyltransferase (Table 4). This enzyme is typically found in Gram-positive bacteria and is involved in the biosynthesis of polyglycerol phosphate teichoic acids, which play a vital function in the rigidity and porosity of bacterial cell walls and biofilm production [31].

### 3.3. Antimicrobial Resistance and Pathogenicity

Several Antibiotic-Resistant Organism (ARO) terms were identified within the four Indonesian skin microbiome-derived bacterial genomes by CARD and ResFinder (Table 5). *B. subtilis* MBF10-19J harbors sixteen putative antimicrobial resistance genes that could confer resistance to streptomycin and tetracycline, benzalkonium chloride, and vancomycin. Only one putative antibiotic resistance gene was found in *M. luteus* MBF05-19J, which relates to glycopeptide resistance. *S. hominis* MBF12-19J and *S. warneri* MBF02-19J contain six and four resistance genes, respectively, potentially conferring resistance to norfloxacin, acriflavine, and vancomycin. Only *B. subtilis* MBF10-19J and *S. hominis* MBF12-19J were predicted to contain putative beta-lactamase-encoding genes.

Using BAGEL4 [19], the four genome sequences were examined for bacteriocin-encoding loci. Bacteriocins are ribosomally synthesized proteinaceous antimicrobials that kill or inhibit the growth of species (usually) closely related to the producer organism [32]. Only two known bacteriocins, namely subtilosin [33], a class of post-translationally modified peptides consisting of thioether bonds, and warnericin RC [34], a lantibiotic known for its antimicrobial activity against *Legionella pneumophila*, were detected (Table 6). Subtilosin and warnericin RC were detected in *B. subtilis* MBF10-19J and *S. warneri*, MBF02-19J, respectively. No bacteriocin-related genes were detected by BAGEL4 in the genome sequences of *M. luteus* MBF05-19J or *S. hominis* MBF12-19J. Meanwhile, *S. hominis* MBF12-19J and *S. warneri* MBF02-19J each bear one autoinducing peptide, namely 314.1 Auto-Inducing Peptide (AIP) I and 315.1 Auto-Inducing PeptideAIP II, respectively. These peptides are signaling molecules involved in the quorum sensing of bacterial communities.

**Figure 1:**
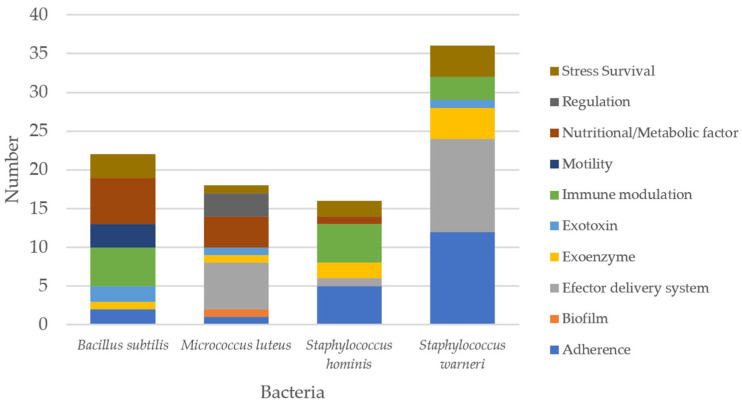
Virulence factors detected in the skin microbiome-derived bacterial strains.

A number of virulence factors were identified from Indonesian skin microbiome bacteria (Figure 1). All bacteria contain virulence factors related to adherence, exoenzyme, and stress survival at various quantities. Meanwhile, genes related to motility were only identified in *B. subtilis* MBF10-19J, and genes related to biofilm formation and regulation were found only in *M. luteus* MBF05-19J. Virulence factors associated with nutritional and metabolic factors were found predominantly in *B. subtilis* MBF10-19J and *M. luteus* MBF05-19J. Meanwhile, virulence genes related to immune modulation were found in *B. subtilis* MBF10-19J, *S. hominis* MBF12-19J, and *S. warneri* MBF02-19J. Putative exotoxin and immune modulation-related genes were found in all strains except for *S. hominis* MBF12-19J and *M. luteus* MBF05-19J, respectively. A detailed list of putative virulence factors is presented in Supplementary Table S2. Although these virulence-associated genes were identified, their expression is not guaranteed under standard laboratory conditions and may depend on specific environmental triggers; therefore, their phenotypic activity needs to be confirmed through targeted in vitro and in vivo analyses.

### 3.4. Toxic Biochemicals

**Figure 2:**
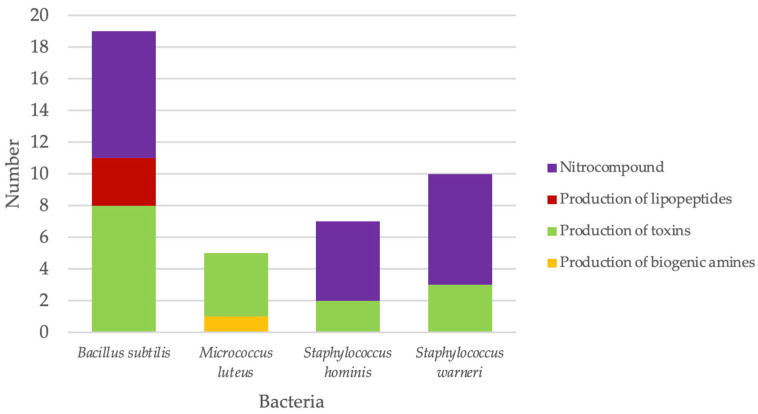
Number and type of genes potentially associated with the production of toxic metabolites.

Genetic loci potentially encoding the production of various toxic biochemicals were identified from all four genome sequences investigated here, Figure 2, Supplementary Table S3), and these were dominated by the hemolysin family protein, which is involved in the host’s cell membrane disruption. *B. subtilis* MBF10-19J, *S. hominis* MBF12-19J, and *S. warneri* MBF02-19J contained genes encoding enzymes directly related to the production of nitrogenous compounds, which are dominated by the nitroreductase family of proteins. Lastly, *B. subtilis* MBF10-19J and *M. luteus* MBF05-19J contain genes associated with the production of lipopeptides and biogenic amines, respectively.

### 3.5. Bacterial Genome Stability

Putative CDSs for CRISPR-Cas sequences were identified in the genome sequences of *B. subtilis* MBF10-19J, *M. luteus* MBF05-19J, and *S. warneri* MBF02-19J. There are three CDSs for CRISPR in *B. subtilis* MBF10-19J and *M. luteus* MBF05-19J, and two CDSs for CRISPR in *S. warneri* MBF02-19J. Each CDS contains various numbers of single associated cas-gene, or repeat consensus, and spacers detected by CRISPRCasMeta (Table 7). No putative CRISPR-Cas sequences were detected in the *S. hominis* MBF12-19J genome sequence.

**Figure 3:**
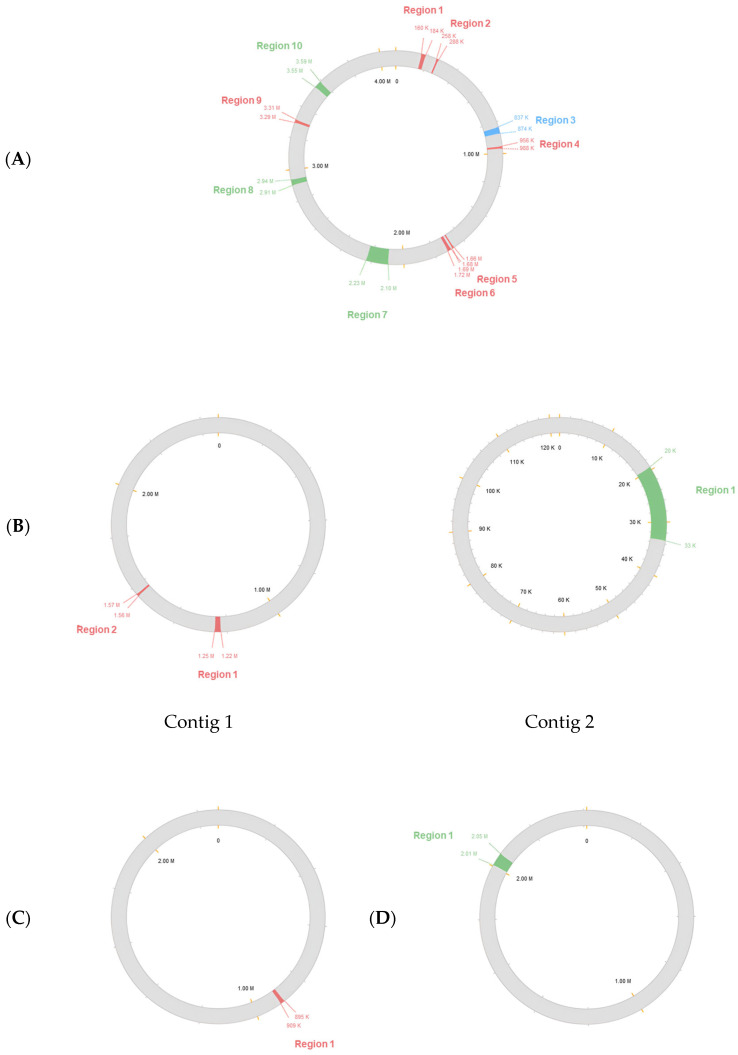
Regions and positions occupied by different types of prophages in circular genome mapping: (**A**) *B. subtilis* MBF10-19J in contig 1; (**B**) *M. luteus* MBF05-19J in contig 1 and 2; (**C**) *S. hominis* MBF12-19J in contig 1; and (**D**) *S. warneri* MBF02-19J in contig 1. Prophage identified as intact (green), incomplete (red), and inconclusive (blue).

Ten predicted prophage regions were identified within the genome of *B. subtilis* MBF10-19J, of which three were classified as intact, six were designated as incomplete, and the remaining region deemed inconclusive. Three putative prophage regions (one intact and two incomplete) were detected in the *M. luteus* MBF05-19J genome. On the other hand, the genomes of *S. hominis* MBF12-19J and *S. warneri* MBF02-19J each contain one incomplete and one apparently intact prophage region, respectively (Figure 3).

A total of twelve insertion sequence (IS) elements were detected within the four genomes (Table 8). Notably, members of the IS*1182*, IS*6*, and IS*3* insertion sequence families were detected, which are related to genomic instability, activation, and/or inactivation of genomic loci and genomic rearrangements, respectively [23].

### 3.6. In Vitro Antibiotic Sensitivity and Antimicrobial Activity Assays

In order to determine whether the ARO Terms (genotypes) identified by CARD and ResFinder correlated with actual antimicrobial resistance phenotypes, disk diffusion and MIC assays were performed according to CLSI guidelines with six clinically relevant antibiotics (vancomycin, gentamicin, erythromycin, chloramphenicol, ciprofloxacin, and amoxicillin). Inhibition zones were interpreted according to available CLSI breakpoints [24,35]. With the exception of *B. subtilis* MBF10-19J, which exhibited resistance to amoxicillin, all four strains were susceptible to all the antibiotics tested (Table 9).

According to CLSI guidelines, antibiotic susceptibility assessment for *Staphylococcus* spp. should be performed using the MIC microdilution method. Both the genomes of *S. warneri* MBF02-19J and *S. hominis* MBF12-19J harbored quinolone-associated genes (*sdrM* and *gyrB*) and vancomycin resistance-associated genes within the *vanG* cluster (*vanT* and/or *vanY*). Despite the presence of these genes, MIC testing showed susceptibility to ciprofloxacin in both strains. *S. hominis* MBF12-19J was susceptible to vancomycin, while *S. warneri* MBF02-19J exhibited intermediate susceptibility (Table 10). In addition, *S. hominis* MBF12-19J carried a *blaZ* (beta-lactamase) gene but remained susceptible to amoxicillin. Collectively, these results indicate that the detected determinants were non-functional.

For *B. subtilis* MBF10-19J and *M. luteus* MBF05-19J, for which limited CLSI breakpoints are available, both disk diffusion and MIC assays were performed to determine their antibiotic susceptibilities. *B. subtilis* MBF10-19J carried genes associated with multidrug resistance, including *ykkC*, *ykkD*, and *mphK*, yet remained susceptible to both chloramphenicol and erythromycin by disk diffusion and MIC testing. In contrast, *B. subtilis* MBF10-19J exhibited resistance to amoxicillin, consistent with the detection of β-lactamase-encoding genes (*blaZ*). Several vancomycin resistance-associated genes were identified in *B. subtilis* MBF10-19J (*vanY* in the *vanM* cluster, *vanW* in the *vanI* cluster, and *vanT* in the *vanG* cluster); however, MIC testing indicated susceptibility to vancomycin, indicating that these *van* clusters were either incomplete or non-functional.

In the case of *M. luteus* MBF05-19J, the only antibiotic resistance-associated gene detected in its genome was a putative *vanY* within the *vanA* cluster. Due to the absence of CLSI breakpoint guidelines for the genus *Micrococcus*, we decided to apply conservative susceptibility breakpoint values, i.e., specifically those that apply to the genus *Staphylococcus*, based on the fact that *Micrococcus* and *Staphylococcus* are Gram-positive genera with similar cell characteristics and habitat preferences (e.g., skin). As shown in Table 9 and Table 10, *M. luteus* MBF05-19J exhibits intermediate susceptibility to vancomycin by MIC and susceptibility by disk diffusion, and is susceptible to all other antibiotics tested by disk diffusion. A summary comparison between in silico predictions and in vitro phenotypic results is provided in Supplementary Table S4.

All of the skin microbiome isolates demonstrated various degrees of antimicrobial activity against Gram-positive and Gram-negative bacteria (Table 11). *B. subtilis* MBF10-19J exhibited strong activity, producing inhibition zones up to 2.37 mm, particularly against *P. aeruginosa* and *E. coli*. *S. warneri*, showed zones ranging from 2.00 to 2.30 mm across all test pathogens, while *S. hominis* MBF12-19J produced slightly larger inhibition zones between 2.17 and 2.37 mm. *M. luteus* MBF05-19J also inhibited all test bacteria, with inhibition zones ranging from 1.88 to 2.23 mm. Overall, the four skin microbiome isolates displayed comparable antimicrobial activity, with minor variations between Gram-positive and Gram-negative bacteria. The correlation between the in silico prediction and the in vitro observation is presented in Supplementary Table S4.

## 4. Discussion

The investigation of the diversity and functions of the human skin microbiome has gained considerable attention in dermatological research. The utilization of skin-dwelling bacteria and their derived metabolites for probiotic applications emerges as a promising avenue for developing innovative microbiome-based ingredients for cosmetics or promoting skin health. However, to be effective probiotics, candidate strains must display important beneficial probiotic-related genes and exhibit genomic stability. Moreover, to be granted “generally regarded as safe” (GRAS) status, probiotic candidates must not either (i) contain complete genetic determinants that confer antibiotic resistance to the host (or be transferable to other hosts) or (ii) encode known virulence factors. In our previous studies, we have reported the isolation of four bacterial strains, *M. luteus* MBF05-19J, *B. subtilis* MBF10-19J, *S. warneri* MBF02-19J, and *S. hominis* MBF12-19J, and their complementary behavior when incorporated into a bacterial cocktail [4,6]. Here, we present the bioinformatic analyses of the genome sequences of these probiotic candidates, focusing on potentially beneficial genes/genetic loci and assessing their features with criteria typically considered in GRAS evaluations, as summarized in Table 12.

The presence of potentially beneficial probiotic-related genes, including those responsible for lactic acid production, adhesion, and stress tolerance, was detected in the genomes of all four strains investigated here. Previous studies have shown that lactic acid can reduce melanin synthesis, resulting in a skin-whitening effect. This occurs through several mechanisms, such as inhibiting the expression of tyrosinase, the key enzyme in melanin biosynthesis, or interfering with tyrosinase glycosylation [36]. In addition, another study has shown that increased acidity of the environment occupied by lactic acid bacteria may inhibit the growth of neighboring pathogens and also inactivates the human immunodeficiency virus [37].

Effective skin probiotics must also persist on the skin surface. This allows the skin microbiome to deliver beneficial effects to target sites, reduces wash-off by skin cleansing products, and maintains their presence and activity at the intended skin surface site [38]. The potential rigidity and porosity of cell walls and biofilm production properties were also exhibited by all strains [39]. While excessive biofilm production may cause skin irritation and inflammation, the ability to construct biofilm may help the probiotic adhere more strongly to the skin surface and survive environmental stresses, such as washing and chemical exposure. Further research is necessary to confirm the activity and potential benefit of the genes identified in our four strains for use in skin-care products.

The genomic stability properties represented by the CRISPR-Cas sequence serve as a defense mechanism against any incoming foreign DNA, such as plasmids, insertion sequences, and bacteriophages. *M. luteus* MBF05-19J, *B. subtilis* MBF10-19J, and *S. warneri* MBF02-19J harbor CRISPR-Cas sequences, suggesting an enhanced capacity to restrict the acquisition of foreign DNA, including antimicrobial resistance genes [40]. On the other hand, these strains also contain prophages and IS elements, which are usually associated with genomic instability, genomic activation/inactivation, rearrangement, and internal plasticity [41]. The coexistence of CRISPR-Cas systems, prophages, and IS elements reflects a balance between genome defense and adaptive flexibility, potentially contributing to niche adaptation without necessarily increasing antimicrobial resistance gene acquisition. For the four strains investigated here, future studies can be carried out to assess genomic stability, e.g., the intra-chromosomal movement/excision of IS elements or prophages over time.

In addition, profiling the properties that are consistently found in those species/strains that have achieved GRAS status is essential; this covers the presence of (potentially) transferable AMR genes and potential virulence factor (VF)-encoding genes. *M. luteus* MBF05-19J was identified as the only bacterium that carried one AMR gene. Although antimicrobial resistance genes are harmful and must be monitored in probiotic screening, their presence does not necessarily prevent these bacteria from obtaining GRAS status [42]. Indeed, *M. luteus* MBF05-19J proved sensitive to the range of clinically relevant antibiotics tested here. The presence of intact prophage regions in *B. subtilis* MBF10-19J, *M. luteus* MBF05-19J, and *S. warneri* MBF02-19J may contribute to horizontal gene transfer and lysogenic conversion, such as a risk of the spread of antibiotic resistance and other virulence factors among bacteria. Several virulence factors were identified in the genomes of all four strains, i.e., factors related to adherence, exoenzyme, immune modulation, motility, biofilm formation, and stress survival. However, such so-called “virulence factors” function mainly in colonization and establishment within a particular habitat and are also typically found in common skin commensals, e.g., *Staphylococcus epidermidis* and *Cutibacterium acnes*. Therefore, these factors should not be considered as classical virulence determinants, i.e., major toxins, superantigens, or invasion-associated systems typically linked to *bona fide* pathogenic bacteria [43,44]. Further in vitro and in vivo investigations are required to assess their potential impact on human skin health and the risk of horizontal gene transfer of these resistance genes to other members of the skin microbiome.

The antibiotic resistance profiles inferred from the in silico genomic analyses were not corroborated by the in vitro susceptibility testing (phenotypic) data. In *Staphylococcus* spp., genes associated with quinolone and vancomycin resistance were detected; however, these determinants alone do not confer phenotypic resistance in the absence of key mutations or regulatory operons, such as *gyrA*/*parC* mutations for fluoroquinolones and the *vanA* operon for glycopeptide (vancomycin) resistance [45,46]. Indeed, both *S. warneri* MBF02-19J and *S. hominis* MBF12-19J were susceptible to ciprofloxacin and vancomycin. Similarly, the presence of *blaZ* in *S. hominis* MBF12-19J did not translate into amoxicillin resistance, consistent with previous reports linking specific *blaZ* polymorphisms to reduced β-lactamase activity [47].

*B. subtilis* MBF10-19J also demonstrated different results across in silico and in vitro analyses. Genes encoding putative multidrug efflux pumps and macrolide-modifying enzymes were detected, yet chloramphenicol and erythromycin susceptibility was maintained. This is in line with previous work demonstrating that *mphK* exhibits poor phosphorylation of erythromycin, resulting in a susceptible phenotype [48]. In contrast, the in vitro result for amoxicillin resistance was consistent with the detection of *blaZ* in the MBF10-19J genome [49]. Vancomycin resistance genes in *B. subtilis* MBF10-19J and *M. luteus* MBF05-19J were also identified by in silico analyses. However, none represented the key regulatory components required for high-level resistance, which was supported by phenotypic susceptibility testing [50]. As no validated CLSI breakpoints are available for *B. subtilis* and *M. luteus*, this study reports MIC and disk diffusion data, prioritizing MIC values for quantitative precision over qualitative zone interpretations [50,51,52].

The in vitro analyses were performed to validate phenotypic expression of the genotypes identified through in silico prediction. However, these assays alone are insufficient to determine GRAS acceptance or substantiate probiotic efficacy and long-term safety. Notably, these assays do not capture gene expression dynamics, host–microbe interactions, or the community-level effects that are critical for assessing probiotic efficacy and long-term safety on the skin. Therefore, comprehensive evaluation will require additional mechanistic, host-relevant, and longitudinal studies beyond standard antimicrobial susceptibility testing.

Although the majority of lactic acid bacteria are known to produce bacteriocins, no bona fide bacteriocin-related genes were identified in *M. luteus* MBF05-19J or *S. hominis* MBF12-19J. In contrast, biosynthetic genes encoding subtilosin in *B. subtilis* MBF10-19J and warnericin RC in *S. warneri* MBF02-19J were detected. These bacteriocins may be beneficial for their antimicrobial activity against closely related bacterial taxa. In the context of the skin microbiome, such activity may be beneficial by limiting opportunistic pathogens such as *Staphylococcus epidermidis*; however, it poses the risk of disturbing the skin microbiome ecosystem by suppressing other commensal species. As the ecological consequences of bacteriocin production on native skin microbial communities remain unknown, further functional and ecological studies are required to clarify the roles of these bacteriocins in microbiome stability.

In vitro antimicrobial screening of *B. subtilis* MBF10-19J and *M. luteus* MBF05-19J both demonstrated positive activity against Gram-positive and Gram-negative bacteria. Despite the absence of bacteriocin-related genes, *M. luteus* is known to produce carotenoid pigments that exhibit antimicrobial activity. This activity has been demonstrated in pigment extract preparations, while antimicrobial activity from intact cells has not been clearly documented [53]. In another study, subtilosin from *B. subtilis* had shown inhibition against Gram-positive bacteria, particularly *Listeria* spp. Although this study did not include *Listeria* spp., *B. subtilis* nonetheless showed inhibitory activity against other Gram-positive bacteria, indicating that its antimicrobial mechanism remained active under the tested conditions [54].

Potential quorum sensing of bacterial communities was assumed to be carried by strains *S. hominis* MBF12-19J and *S. warneri* MBF02-19J as the peptide signaling molecules were identified, i.e., 314.1 AIP I and 315.1 AIP II, respectively. AIP in Gram-positive bacteria ranging from 5 to 17 of linear or cyclized amino acids. It allows bacteria to share information and adjust gene expression in response to the environment or on a host. A previous study showed that the presence of a quorum sensing system in a bacterial community regulates the expression of genes encoding virulence factors in *S. aureus* found in human skin flora [37]. Therefore, the presence of genes related to quorum sensing in our four skin microbiome strains could prove a benefit for these strains to be used as components of a bacterial cocktail for promoting skin health. The in vitro antimicrobial assay supported the in silico prediction that both *S. hominis* MBF12-19J and *S. warneri* MBF02-19J exhibited inhibitory activity against Gram-positive and Gram-negative bacteria.

Genes involved in toxin production were identified from all strains, and these were dominated by the hemolysin family. *B. subtilis* MBF10-19J, *S. hominis* MBF12-19J, and *S. warneri* MBF02-19J harbored genes encoding enzymes directly related to the production of nitro compounds, which are dominated by the nitroreductase family. In addition, *B. subtilis* MBF10-19J and *M. luteus* MBF05-19J contain genes associated with the production of lipopeptides and biogenic amines, respectively. The presence of these gene categories indicates a genetic potential for producing metabolites that could be harmful under certain conditions.

Notably, previous skin sensitivity tests using a bacterial cocktail of these strains did not reveal any adverse reaction, suggesting an absence of any obvious short-term toxicity [5]. However, the detection of genes related to hemolysins, nitroreductases, and other potentially bioactive metabolites warrants cautious interpretation, as a genetic presence does not necessarily reflect expression levels or metabolite production in situ. Moreover, the regulation of these pathways may be influenced by host-specific factors, environmental cues, or prolonged exposure, which are not captured by the present in silico analysis. Therefore, while the available data support short-term skin compatibility, additional functional assays and long-term exposure studies are required to more comprehensively assess safety.

While a gap exists between genotype and phenotype, it is important to recognize that phenotype is a manifestation of an organism’s genotype under specific (i.e., in vivo) conditions. Therefore, assessing bacterial safety which aligns and complies with existing GRAS evaluation frameworks through whole-genome analysis provides an important foundation for understanding the potential risks and benefits of probiotic candidates. This approach can contribute to addressing the challenging safety assessment process and the rising concerns about the potential risks of probiotic products on human health.

## 5. Conclusions

Each skin microbiome bacterial strain investigated here harbors potential beneficial probiotic properties, such as lactic acid biosynthesis, cell adhesion, and metabolism improvement. On the other hand, whilst several undesirable genetic elements, such as those related to antimicrobial resistance, pathogenicity, and toxic biochemical, were also detected, functionality with respect to antibiotic resistance was not substantiated by in vitro testing. Additional information from this study supports the potential of the four skin microbiome-derived strains analyzed here as skin probiotic candidates. In vitro analyses indicate that *S. hominis* MBF12-19J is suitable as a probiotic. *S. warneri* MBF02-19J and *M. luteus* MBF05-19J appear safe for probiotic applications when used with appropriate caution, given the lack of validated AMR breakpoints for *M. luteus*. Whilst *B. subtilis* MBF10-19J displays amoxicillin resistance, it remains susceptible to other commonly used antibiotics such as erythromycin. Further in vivo work, including animal models and human trials, is warranted to comprehensively evaluate strain activities and safety under physiological conditions.

## Figures and Tables

**Table 1:** Assembled genome characteristics.

Genome Assembly	*B. subtilis* MBF10-19J	*M. luteus* MBF05-19J	*S. hominis* MBF12-19J	*S. warneri* MBF02-19J
Genome size (bp)	4,124,541	2,588,352	2,295,496	2,521,958
Number of contigs	2	2	2	12
Contig N50 (bp)	4,099,904	2,466,513	2,270,862	2,412,201
GC percent (%)	43.5	73.0	31.5	33.0
Genome coverage	275.0×	250.0×	230.0×	275.0×
Genes	4390	2424	2214	2466
Protein-coding	3919	2155	2037	2328
Completeness (%) ^1^	91.25	89.93	89.28	99.65
Contamination (%) ^1^	0.92	0.46	5.12	0.06

^1^ Quality analysis was calculated using CheckM with corresponding bacterial marker set [28].

**Table 2:** Putative protein-encoding genes associated with lactic acid production.

Bacteria	Gene Function	Contig No.	Nucleotide Position	Protein Length (aa)	Orientation
Bacteria*B. subtilis* MBF10-19J	L-lactate dehydrogenase	1	3933240–3934205	321	−
*M. luteus* MBF05-19J	L-lactate dehydrogenase	1	2366161–2367147	328	−
*S. hominis* MBF12-19J	L-lactate dehydrogenase	1	595506–596468	320	−
	D-lactate dehydrogenase	1	566226–567224	332	+
	D-lactate dehydrogenase	1	612912–613904	330	−
*S. warneri* MBF02-19J	L-lactate dehydrogenase	1	676931–677887	318	+
	D-lactate dehydrogenase	1	710609–711607	332	+
	D-lactate dehydrogenase	1	752237–753229	330	−

**Table 3:** Identified protein-encoding genes putative for adhesion.

Bacteria	Gene Function	Contig No.	Nucleotide Position	Protein Length (aa)	Orientation
*B. subtilis* MBF10-19J	Tyrosine-protein kinase PtkA	1	641786–642499	237	+
*M. luteus* MBF05-19J	Class F sortase	1	1154404–1155192	262	−
	Tyrosine-protein kinase family protein	1	651873–653456	527	+
*S. hominis* MBF12-19J	Class A sortase SrtA	1	611182–611835	217	+
	Tyrosine-protein kinase	1	484252–484959	235	+
*S. warneri* MBF02-19J	Class A sortase SrtA	1	750585–751199	204	+

**Table 4:** Identified protein-encoding genes involved in active metabolism.

Bacteria	Gene Function	Contig No.	Nucleotide Position	Protein Length (aa)	Orientation
*S. hominis* MBF12-19J	poly(glycerol-phosphate) alpha-glucosyltransferase	1	2212230–2213852	540	−
*S. warneri* MBF02-19J	poly(glycerol-phosphate) alpha-glucosyltransferase	1	113739–115352	537	−

**Table 5:** Putative antimicrobial resistance (AMR) genes detected.

Bacteria	ARO Term	AMR Gene Family	Antibiotic	Contig No.	Nucleotide Position	Orientation
*B. subtilis* MBF10-19J	*B. subtilis mprF*	Defensin resistant *mprF*	Defensin	1	3311549–3314119	−
	*bmr*	Major facilitator superfamily (MFS) antibiotic efflux pump	Acriflavine; puromycin; chloramphenicol	1	1868867–1870036	−
	*lmrB*	ATP-binding cassette (ABC) antibiotic efflux pump	Lincomycin; puromycin	1	3977015–3978448	+
	*ykkC*	Small multidrug resistance (SMR) antibiotic efflux pump	Streptomycin; tetracycline; chloramphenicol	1	2876690–2877028	−
	*ykkD*	SMR antibiotic efflux pump	Streptomycin; tetracycline; chloramphenicol	1	2876373–2876690	−
	*vmlR*	Miscellaneous ABC-F subfamily ATP-binding cassette ribosomal protection proteins	Lincomycin; virginiamycin M1; Tiamulin; virginiamycin S2; retapamulin; iboxamycin; hygromycin A; A201A	1	3637619–3639262	+
	*tmrB*	Tunicamycin resistance protein	Tunicamycin	1	3900254–3900847	+
	*mphK*	Macrolide phosphotransferase (MPH)	Telithromycin; azithromycin; spiramycin	1	3987522–3988436	−
	FosBx1	Fosfomycin thiol transferase	Fosfomycin	1	2395072–2395506	−
	*qacJ*	SMR antibiotic efflux pump	Benzalkonium chloride	1	2440760–2441080	+
	*qacJ*	SMR antibiotic efflux pump	Benzalkonium chloride	1	2441094–2441447	+
	*qacG*	SMR antibiotic efflux pump	Benzalkonium chloride	1	967469–967828	+
	*vanW* gene in *vanI* cluster	Vancomycin resistance gene cluster; *vanW*	Vancomycin; teicoplanin	1	2286798–2287709	+
	*vanY* gene in *vanM* cluster	Vancomycin resistance gene cluster; *vanY*	Vancomycin; teicoplanin	1	2222363–2223184	+
	*vanT* gene in *vanG* cluster	Vancomycin resistance gene cluster; *vanT*	Vancomycin	1	3727051–3728220	−
	PC1 beta-lactamase (*blaZ*)	BlaZ beta-lactamase	Amoxicillin; ampicillin; piperacillin; penicillin	6	8830–8958	−
*M. luteus* MBF05-19J	*vanY* gene in *vanA* cluster	Vancomycin resistance gene cluster; *vanY*	Vancomycin; teicoplanin	1	418177–418926	−
*S. hominis* MBF12-19J	*fusC*	Steroid antibacterial; target protecting FusB-type protein conferring resistance to fusidic acid	Fusidic acid	1	210712–211350	+
	*sdrM*	MFS antibiotic efflux pump	Norfloxacin	1	933792–935132	+
	*sepA*	SMR antibiotic efflux pump	Acriflavine	1	935207–935674	+
	*vanT* gene in *vanG* cluster	Vancomycin resistance gene cluster; *vanT*	Vancomycin	1	1016536–1017681	+
	*vanY* gene in *vanG* cluster	Vancomycin resistance gene cluster; *vanY*	Vancomycin	1	288086–288757	−
	PC1 *blaZ*	BlaZ beta-lactamase	Amoxicillin; ampicillin; piperacillin; penicillin	2	18741–18869	+
*S. warneri* MBF02-19J	*S. aureus gyrB* conferring resistance to aminocoumarin	Aminocoumarin resistance *gyrB*	Novobiocin; clorobiocin; couMermycin A1	1	384334–386256	+
	*sdrM*	MFS antibiotic efflux pump	Norfloxacin	1	1100070–1101407	+
	*sepA*	SMR antibiotic efflux pump	Acriflavine	1	1101527–1101994	+
	*vanT* gene in *vanG* cluster	Vancomycin resistance gene cluster; *vanT*	Vancomycin	1	1189839–1190987	+

**Table 6:** Identified bacteriocin genes within the four skin microbiome-derived bacterial genomes.

Bacteria	Amino Acid Sequence	Class	Subclass	Contig	Nucleotide Position
*B. subtilis* MBF10-19J	LKLPVQQVYSVYGGKDLPKGHSHS TMPFLSKLQFLTKIYLLDIHTQPFFI	216.2; Subtilosin (SboX)	Sactipeptide	1	536282–556896
*S. hominis* MBF12-19J	MTFITQLFIKLFSLILETVGTLASYSP CATYFDEPEVPEELTNLER	314.1; AIP I	Auto Inducing Peptides	1	1042904–1063039
*S. warneri* MBF02-19J	MQFITDLIKKAVDFFKGLFGNK	226.2; warnericin RC	-	1	1343093–1363156
	MEFLVNLFFKFFTSIMEFVGFVAGYS PCTNFFDEPEVPSE LTKIYE	315.1; AIP II	Auto Inducing Peptides	1	1219862–1240957

**Table 7:** Putative CRISPR-Cas sequences detected in the skin microbiome bacterial genome sequences.

Bacteria	Repeat Consensus/Cas-Genes	Contig No.	Nucleotide Position	Spacer Count
*B. subtilis* MBF10-19J	ATCAATCATCCAAATCTGGTCGTTCGTCA ATCAATCATCAAAATCATACAGCTCATCA ATCAATCATCAAAATCATACAGCTCATCA ATCAATCATCAAGATCATCAGGTTATTCA	1	657275–657546	3
	AGAAGAGCTTGCTGTGCCGGAAAAGGAGGTTCGTGCTGAATCGG AGAAGAGCTTGCTGTGCCGGAAAAGGAGGTTCGTGCTGAATCGG AGAAGAGCTTGCTGTGCCGGAAAAGGAGGTTCGTGCTGAATCGG AGAAGAGCTTGCTGTGCCGGAAAAGGAGGTTCGTGCTGAATCGG AGAAGAGCTTGCTGTGCCGGAAAAGGAGGTTCGTGCTGAATCGG AGAAGAGCTTGCTGTGCCGGAAAAGGAGGTTCGTGCTGAATCGG AGAAGAGCTTGCTGTGCCGGAAAAGGAGGTTCGTGCTGAATCGG AGAAGAGCTTGCTGTGCCGGAAAAGGAGGTTCGTGCTGAATCGG	1	1558676–1559556	9
	TCTTGATAGAACTCTTTGTCATGATT TCTTGATAGAATTCTTTGTCATGGTT TCTTGATAGAATTCTTTGTCATGGTT	1	3752233–3752378	2
*M. luteus* MBF05-19J	CCTGACCGCGGCCCAGCTCGAGG CCCGACCCGCGCCGAGCGCGACG CAAGGACCGCGGCGAGCGCGGCG CAAGGACCGGGACGACCGCGGCT CAAGGACCGGGACGACCGCGGCT CTCGGATCGGGGCGCCCGCCGCT	1	359137–359495	5
	AGTTCTGACGCCCGATCCGCAGCG AGTTCTGACGCCCGATCCGCAGCG	1	2345977–2346071	1
	CTGGCTCATCCCTGCGCGGGCGGAGCTTCC TGGGCTCATCCCTGCGTGCGCGGGGCTTCC TGGGCTCATCCCTGCGTGCGCGGGGCTTCC	2	15525–15676	2
*S. warneri* MBF02-19J	AAGTACTTCCATTTTAATGGTTAG AAGTACTTCCATTTTAATGGTTAG	1	885832–885918	1
	TTAAAGGCATAGTTTTTTTGTTGTTATGCCT TTAAAGGCATAGTTTTTTTGTTGTTATGCCT	13	2145–2257	1

**Table 8:** Identified bacterial insertion sequences found within the four skin microbiome-derived bacterial genomes.

Bacteria	Sequences Producing Significant Alignments	IS Family	Origin	Score	E Value
*B. subtilis* MBF10-19J	ISBwe2	IS*6*	*Bacillus weihenstephanensis*	303	1.00 × 10^−78^
	IS643	IS*21*	*Bacillus halodurans*	266	3.00 × 10^−67^
	ISBwe3	IS*6*	*Bacillus weihenstephanensis*	242	4.00 × 10^−60^
	IS240C	IS*6*	*Bacillus cereus*	214	9.00 × 10^−52^
	ISBsp5	IS*1182*	*Bacillus* sp.	143	3.00 × 10^−30^
	ISBth6	IS*6*	*Bacillus thuringiensis*	143	3.00 × 10^−30^
	ISOih1	IS*1182*	*Oceanobacillus iheyensis*	123	3.00 × 10^−24^
	ISBpu1	IS*1182*	*Bacillus pumilus*	119	4.00 × 10^−23^
	IS240B	IS*6*	*Bacillus thuringiensis*	115	6.00 × 10^−22^
	IS240A	IS*6*	*Bacillus thuringiensis*	115	6.00 × 10^−22^
	ISBspe1	IS*1182*	*Bacillus pseudofirmus*	89.7	4.00 × 10^−14^
*M. luteus* MBF05-19J	ISPfr10	IS*3*	*Propionibacterium freudenreichii*	1279	0
	ISPfr12	IS*3*	*Propionibacterium freudenreichii*	1251	0
	ISAar43	IS*3*	*Arthrobacter arilaitensis*	1237	0
	ISBli29	IS*NCY*	*Brevibacterium linens*	414	3.00 × 10^−112^
	ISArsp9	IS*NCY*	*Arthrobacter* sp.	315	2.00 × 10^−82^
	ISBli17	IS*3*	*Brevibacterium linens*	186	1.00 × 10^−43^
	ISTesp1	IS*3*	*Terrabacter* sp.	180	8.00 × 10^−42^
	ISBli35	IS*3*	*Brevibacterium linens*	157	1.00 × 10^−34^
	ISArsp6	Tn*3*	*Arthrobacter* sp.	147	1.00 × 10^−31^
	ISArsp14	IS*NCY*	*Arthrobacter* sp.	137	1.00 × 10^−28^
	ISAcl2	IS*3*	*Arthrobacter chlorophenolicus*	137	1.00 × 10^−28^
	ISMyma1	IS*3*	*Mycobacterium marinum*	135	4.00 × 10^−28^
	ISMcte1	IS*5*	*Micrococcus terreus*	103	1.00 × 10^−18^
	ISRhosp5	IS*3*	*Rhodococcus* sp.	101	6.00 × 10^−18^
	ISTesp3	IS*3*	*Terrabacter* sp.	99.6	2.00 × 10^−17^
	IS999	IS*3*	*Mycobacterium avium*	95.6	3.00 × 10^−16^
	ISBli33	IS*3*	*Brevibacterium linens*	81.8	5.00 × 10^−12^
	ISRsp12	Tn*3*	*Rhizhobium* sp.	81.8	5.00 × 10^−12^
	ISPfr13	IS*3*	*Propionibacterium freudenreichii*	81.8	5.00 × 10^−12^
	ISShes11	Tn*3*	*Shewanella* sp.	79.8	2.00 × 10^−11^
	ISAcba1	IS*1595*	*Actinobacteria bacterium*	77.8	8.00 × 10^−11^
*S. hominis* MBF12-19J	ISSep1	IS*1182*	*Staphylococcus epidermidis*	3027	0
	IS1272	IS*1182*	*Staphylococcus haemolyticus*	2510	0
	ISSau3	IS*1182*	*S. aureus*	1065	0
	ISSau4	IS*3*	*S. aureus*	149	2.00 × 10^−32^
	ISCpe5	IS*1182*	*Clostridium perfringens*	81.8	5.00 × 10^−12^
	ISSmi2	IS*1182*	*Streptococcus mitis*	75.8	3.00 × 10^−10^
*S. warner*i MBF02-19J	IS257R1	IS*6*	*S. aureus*	1550	0
	IS431mec	IS*6*	*S. aureus*	1518	0
	IS257R2	IS*6*	*S. aureus*	1511	0
	IS431R	IS*6*	*S. aureus*	1507	0
	IS431L	IS*6*	*S. aureus*	1473	0
	IS257-3	IS*6*	*S. aureus*	1344	0
	IS257-1	IS*6*	*S. aureus*	1322	0
	IS257-2	IS*6*	*S. aureus*	767	0
	ISSau6	IS*6*	*S. aureus*	593	6.00 × 10^−166^
	ISSau3	IS*1182*	*S. aureus*	482	1.00 × 10^−132^
	ISSep1	IS*1182*	*Staphylococcus epidermidis*	367	6.00 × 10^−98^
	IS1272	IS*1182*	*Staphylococcus haemolyticus*	343	9.00 × 10^−91^
	ISSep2	IS*110*	*Staphylococcus epidermidis*	139	3.00 × 10^−29^
	ISSau4	IS*3*	*S. aureus*	131	6.00 × 10^−27^

**Table 9:** Antibiotic susceptibility disk diffusion assays.

Strain	Disk Diffusion (in mm)				
	*S. warneri* MBF02-19J	*S. hominis* MBF12-19J	*B. subtilis* MBF10-19J	*M. luteus* MBF05-19J	Interpretation
Vancomycin	28.72	28.87	23.93	26.02	Susceptible
Gentamicin	34.58	33.05	33.12	35.60	Susceptible
Chloramphenicol	35.65	30.30	34.33	24.87	Susceptible
Erythromycin	24.03	32.52	30.43	35.60	Susceptible
Ciprofloxacin	39.48	44.05	42.65	34.75	Susceptible
Amoxicillin	48.15	32.53	8.02	43.60	Susceptible, except *B. subtilis* MBF10-19J

**Table 10:** Minimum inhibitory concentration (MIC) values.

Strain	Vancomycin (µg/mL)	Interpretation
*S. warneri* MBF02-19J	8	Intermediate
*S. hominis* MBF12-19J	4	Susceptible
*B. subtilis* MBF10-19J	32	Resistant
*M. luteus* MBF05-19J	8	Intermediate
Strain	Amoxicillin (µg/mL)	Interpretation
*B. subtilis* MBF10-19J	50	Resistant

**Table 11:** Antimicrobial activity exhibited by the four skin microbiome-derived bacterial strains.

	*S. warneri* MBF02-19J	*S. hominis* MBF12-19J	*B. subtilis* MBF10-19J	*M. luteus* MBF05-19J
*S. aureus*	2.30|2.27	2.37|2.27	2.37|2.45	2.17|2.23
*B. subtilis*	2.02|2.00	2.17|2.20	2.15|2.10	1.90|1.88
*S. mutans*	2.25|2.12	2.22|2.25	2.37|2.25	2.07|2.05
*S. typhimurium*	2.07|2.17	2.27|2.35	2.20|2.30	2.10|2.15
*P. aeruginosa*	2.15|2.17	2.32|2.37	2.32|2.47	2.07|2.17
*E. coli*	2.20|2.12	2.30|2.25	2.37|2.46	2.17|2.12

Units are in mm (left|right of the center producer streak).

**Table 12:** Key safety acceptability criteria.

Safety Criteria	Acceptability Description
Strain identification	Genus and species of the probiotic strain must be identified and show specific health effect. Recommended methods, i.e., sequencing the 16S rRNA gene amplified by polymerase chain reaction (PCR) and/or whole-genome sequencing (WGS).
Adhesion ability	The genome must be screened for adhesion-related genes to confirm the strain’s ability for colonizing human epithelial cells and interacting with the host immune system as a key functional safety requirement for effective probiotics.
Biofilm formation and antimicrobial ability	The genes related to biofilm formation and bacteriocin production must be identified. Biofilm formation should be characterized with in vitro and in vivo studies to prove non-pathogenicity, while antimicrobial gene presence supports the strain’s role in inhibiting the growth of potential pathogens.
Absence of gene transfer potential	The strain must not contain transferable antibiotic resistance gene to prevent acquired antibiotic resistance (AMR). Ideally, in silico screening of the genome sequence of the strain of interest should be performed.

## Data Availability

The whole-genome sequences of *B. subtilis* MBF10-19J, *M. luteus* MBF05-19J, *S. hominis* MBF12-19J, and *S. warneri* MBF02-19J have been deposited in GenBank under accession numbers JAGMTL000000000, JAGMUB000000000, JAGMUC000000000, and JAGMUP000000000, respectively.

## Supplementary Materials

The following supporting information can be downloaded at: https://www.mdpi.com/article/10.3390/microorganisms14010248/s1, Supplementary Table S1: Raw genome characteristics of the four sequenced bacterial genomes; Supplementary Table S2: Virulence factor-related genes; Supplementary Table S3: Toxic metabolite genes; and Supplementary Table S4: In vitro antibiotic resistance and antimicrobial activity profiles of the four sequenced bacterial genomes.

## Abbreviations

The following abbreviations are used in this manuscript:

AA	Amino acid
ABC	ATP-binding cassette
AIP	Auto-inducing peptides
AMR	Antimicrobial resistance
ARO	Antibiotic-resistant organism
bp	Base pair
CARD	Comprehensive Antibiotic Resistance Database
Cas	CRISPR-associated genes
CDS	Coding sequence
CLSI	Clinical and Laboratory Standards Institute
CRISPR	Clustered regularly interspaced short palindromic repeats
GRAS	Generally recognized as safe
IS	Insertion sequence
MFS	Major facilitator superfamily
MHA	Mueller–Hinton agar
MHB	Mueller–Hinton broth
MIC	Minimum inhibitory concentration
MPH	Macrolide phosphotransferase
PGAP	Prokaryotic Genome Annotation Pipeline
PHASTER	Phage Search Tool Enhanced Release
RAST	Rapid annotations using subsystems technology
RiPPs	Ribosomally synthesized and post-translationally modified peptides
SMR	Small multidrug resistance
VF	Virulence factor
VFDB	Virulence Factor Database
WGS	Whole-genome sequencing
