# Supplementary material for: Genetic Characteristics Associated with Probiotic Functions in Four Indonesian Skin Microbiome-Derived Bacterial Strains
Source: Microorganisms. 2026 Jan 21;14(1):248. doi: 10.3390/microorganisms14010248 (PMC12843939; doi:10.3390/microorganisms14010248)
Supplement: Supplementary file 1 [file microorganisms-14-00248-s001.zip › microorganisms-4075834-supplementary.pdf]

Supplementary Information (Alkaff et al. Microorganisms manuscript ID microorganisms-4075834)

Supplementary Table S1. Raw genome sequence data characteristics of the four sequenced bacterial genomes.

Strain	*Bacillus subtilis* MBF10-19J		*Micrococcus luteus* MBF05-19J		*Staphylococcus hominis* MBF12-19J		*Staphylococcus warneri* MBF02-19J	
QC state	*Before filtering*	*After filtering*	*Before filtering*	*After filtering*	*Before filtering*	*After filtering*	*Before filtering*	*After filtering*
Mean read length (bp)	4,259	11,832	4,287	10,696	5,376	12,577	4,416	12,073
Mean read quality	14.1	16.2	13.5	15.7	12.3	13.9	14.6	16.7
Median read length (bp)	3,622	11,172	3,859	10,065	4,948	11,915	4,024	11,463
Median read quality	14.5	16.2	13.9	15.8	12.6	13.9	15.1	16.7
Number of reads	1,488,420	42,260	1,310,379	46,749	1,052,441	39,757	2,172,057	41,416
Read length N50 (bp)	5,947	11,706	5,862	10,573	7,177	12,491	5,995	11,904
Total sequenced bases	6,339,552,612	500,000,236	5,617,011,117	500,008,485	5,657,931,708	500,010,877	9,592,060,528	500,006,307

Supplementary Table S2. Virulence factor (VF)-related genes.

No	ID	Related VF	Product	Function	Organism
***Bacillus subtilis* MBF10-19J**					
1	VFG000079	*clpC*	endopeptidase Clp ATP-binding chain C	ClpC - Stress survival	*Listeria monocytogenes* EGD-e
2	VFG046465	*tufA*	elongation factor Tu	EF-Tu - Adherence	*Francisella tularensis* subsp. *tularensis* SCHU S4
3	VFG000080	*clpE*	ATP-dependent protease	ClpE - Stress survival	*Listeria monocytogenes* EGD-e
4	VFG000077	*clpP*	ATP-dependent Clp protease proteolytic subunit	ClpP - Stress survival	*Listeria monocytogenes* EGD-e
5	VFG001855	*htpB*	Hsp60, 60K heat shock protein HtpB	Hsp60 - Adherence	*Legionella pneumophila* subsp. *pneumophila* str. Philadelphia 1
6	VFG048830	*gndA*	NADP-dependent phosphogluconate dehydrogenase	Capsule - Immune modulation	*Klebsiella pneumoniae* subsp. *pneumoniae* NTUH-K2044
7	VFG050038	*dhbF*	non-ribosomal peptide synthetase, DhbF	Bacillibactin - Nutritional/Metabolic factor	*Bacillus cereus* ATCC 10987
8	VFG050027	*dhbB*	isochorismatase, DhbB	Bacillibactin - Nutritional / Metabolic factor	*Bacillus cereus* ATCC 10987
9	VFG016767	*fliP*	flagellar biosynthetic protein FliP	Flagella - Motility	*Bartonella bacilliformis* KC583 (ATCC 35685)
10	VFG000682	*capB*	CapB, involved in Poly-gamma-glutamate synthesis	Capsule - Immune modulation	*Bacillus anthracis*
11	VFG013327	*manB/yhxB*	phosphomannomutase	LOS - Immune modulation	*Haemophilus influenzae* Rd KW20
12	VFG002181	*cpsJ*	ABC transporter, ATP-binding protein	Capsule - Immune modulation	*Enterococcus faecalis* V583
13	VFG043366	*tlpA*	membrane-bound chemoreceptor sensing arginine and bicarbonate	Flagella - Motility	*Helicobacter pylori* 26695
14	VFG000925	*fepC*	ferrienterobactin ABC transporter ATPase	Enterobactin - Nutritional/Metabolic factor	*Escherichia coli* CFT073
15	VFG050115	*cesA*	Cereulide synthetase A, CesA	Cereulide - Exotoxin	*Bacillus cereus* AH187
16	VFG007023	*rtxB*	RTX toxin transporter RtxB	MARTX - Exotoxin	*Vibrio cholerae* O1 biovar El Tor str. N16961
17	VFG006607	*fliP*	flagellar biosynthetic protein FliP	Flagella - Motility	*Helicobacter pylori* 26695
18	VFG050016	*dhbE*	2,3-dihydroxybenzoate adenylase DhbE	Bacillibactin - Nutritional/Metabolic factor	*Bacillus cereus* ATCC 10987
19	VFG000680	*capA*	CapA, required for Poly-gamma-glutamate transport	Capsule - Immune modulation	*Bacillus anthracis*

20	VFG048498	*entF*	enterobactin synthase subunit F	Ent - Nutritional/Metabolic factor	*Klebsiella pneumoniae* subsp. *pneumoniae* NTUH-K2044
21	VFG002281	*nagL*	hyaluronidase	Mu-toxin - Exoenzyme	*Clostridium perfringens* str. 13
22	VFG049994	*dhbA*	2,3-dihydroxybenzoate-2,3-dehydrogenase, DhbA	Bacillibactin - Nutritional/Metabolic factor	*Bacillus cereus* ATCC 10987
Micrococcus luteus MBF05-19J					
1	VFG001381	*icl*	Isocitrate lyase Icl (isocitrase) (isocitratase)	Isocitrate lyase - Others	*Mycobacterium tuberculosis* H37Rv
2	VFG001405	*sigA/rpoV*	RNA polymerase sigma factor SigA	SigA - Regulation	*Mycobacterium tuberculosis* H37Rv
3	VFG041020	*tssH*	type VI secretion system ATPase TssH	HSI-2 - Effector delivery system	*Pseudomonas aeruginosa* PAO1
4	VFG014984	*algW*	AlgW protein	Alginate - Biofilm	*Pseudomonas aeruginosa* PAO1
5	VFG001421	*sodA*	superoxide dismutase	SodA - Stress survival	*Mycobacterium tuberculosis* H37Rv
6	VFG038395	*clpB*	type VI secretion system ATPase ClpV1	T6SS - Effector delivery system	*Aeromonas hydrophila* subsp. *hydrophila* ATCC 7966
7	VFG015530	*phzC2*	phenazine biosynthesis protein PhzC	Pyocyanin - Nutritional/Metabolic factor	*Pseudomonas aeruginosa* PAO1
8	VFG015509	*phzC1*	phenazine biosynthesis protein PhzC	Pyocyanin - Nutritional/Metabolic factor	*Pseudomonas aeruginosa* PAO1
9	VFG002480	*tssH-5/clpV*	Clp-type ATPase chaperone protein	T6SS-1 - Effector delivery system	*Burkholderia pseudomallei* K96243
10	VFG041043	*tssH*	type VI secretion system ATPase TssH	HSI-3 - Effector delivery system	*Pseudomonas aeruginosa* PAO1
11	VFG043648	*rpoS*	RNA polymerase sigma factor RpoS	Type IV pili - Adherence	*Pseudomonas aeruginosa* PAO1
12	VFG001206	*fbpC*	iron(III) ABC transporter, ATP-binding protein	FbpABC - Nutritional/Metabolic factor	*Neisseria meningitidis* MC58
13	VFG001412	*sigH*	ECF RNA polymerase sigma factor SigH	SigH - Regulation	*Mycobacterium tuberculosis* H37Rv
14	VFG002076	*clpV1*	type VI secretion system AAA+ family ATPase	HSI-1 - Effector delivery system	*Pseudomonas aeruginosa* PAO1
15	VFG038916	*rtxB*	RTX toxin transporter, ATPase protein	RtxA - Exotoxin	*Aeromonas hydrophila* subsp. *hydrophila* ATCC 7966
16	VFG048488	*fepC*	iron-enterobactin transporter ATP-binding protein	Ent - Nutritional/Metabolic factor	*Klebsiella pneumoniae* subsp. *pneumoniae* NTUH-K2044
17	VFG049904	*clpV/tssH*	Type VI secretion system ATPase ClpV/TssH	T6SS - Effector delivery system	*Shigella sonnei* Ss046
18	VFG001826	*relA*	Probable GTP pyrophosphokinase RelA (ATP:GTP 3'-pyrophosphotransferase) (PPGPP synthetase I) ((P)PPGPP synthetase) (GTP diphosphokinase)	RelA - Regulation	*Mycobacterium tuberculosis* H37Rv
Staphylococcus hominis MBF12-19J					
1	VFG004773	*lip*	triacylglycerol lipase precursor	Lipase - Exoenzyme	*Staphylococcus aureus* subsp. *aureus* MW2
2	VFG000077	*clpP*	ATP-dependent Clp protease proteolytic subunit	ClpP - Stress survival	*Listeria monocytogenes* EGD-e
3	VFG000079	*clpC*	endopeptidase Clp ATP-binding chain C	ClpC - Stress survival	*Listeria monocytogenes* EGD-e
4	VFG046465	*tufA*	elongation factor Tu	EF-Tu - Adherence	*Francisella tularensis* subsp. *tularensis* SCHU S4
5	VFG012095	*groEL*	chaperonin GroEL	GroEL - Adherence	*Clostridium difficile* 630
6	VFG001316	*geh*	glycerol ester hydrolase	Lipase - Exoenzyme	*Staphylococcus aureus* subsp. *aureus* MW2
7	VFG001300	*cap8D*	type 8 capsular polysaccharide synthesis protein Cap8D	Capsule - Immune modulation	*Staphylococcus aureus* subsp. *aureus* MW2
8	VFG001280	*sdrD*	Ser-Asp rich fibrinogen-binding bone sialoprotein-binding protein	SDr - Adherence	*Staphylococcus aureus* subsp. *aureus* MW2
9	VFG002189	*cpsB/cdsA*	phosphatidate cytidylyltransferase	Capsule - Immune modulation	*Enterococcus faecalis* V583
10	VFG037386	*bauE*	ferric siderophore ABC transporter, ATP-binding protein BauE	Acinetobactin - Nutritional/Metabolic factor	*Acinetobacter baumannii* ACICU
11	VFG001298	*cap8B*	type 8 capsular polysaccharide synthesis protein Cap8B	Capsule - Immune modulation	*Staphylococcus aureus* subsp. *aureus* MW2
12	VFG002160	*fbpA*	fibronectin-binding protein	FbpA - Adherence	*Listeria monocytogenes* EGD-e

13	VFG001855	*htpB*	Hsp60, 60K heat shock protein HtpB	Hsp60 - Adherence	*Legionella pneumophila* subsp. *pneumophila* str. Philadelphia 1
14	VFG001950	*Cj1422c*	sugar transferase	Capsule - Immune modulation	*Campylobacter jejuni* subsp. *jejuni* NCTC 11168
15	VFG001949	*Cj1421c*	sugar transferase	Capsule - Immune modulation	*Campylobacter jejuni* subsp. *jejuni* NCTC 11168
16	VFG051098	*clpV/tssH*	type VI secretion system ATPase TssH	T6SS - Effector delivery system	*Acinetobacter baumannii* ACICU
Staphylococcus warneri MBF02-19J					
1	VFG004773	*lip*	triacylglycerol lipase precursor	Lipase - Exoenzyme	*Staphylococcus aureus* subsp. *aureus* MW2
2	VFG001295	*sspB*	staphopain cysteine proteinase SspB	Staphopain - Exoenzyme	*Staphylococcus aureus* subsp. *aureus* MW2
3	VFG000079	*clpC*	endopeptidase Clp ATP-binding chain C	ClpC - Stress survival	*Listeria monocytogenes* EGD-e
4	VFG001296	*sspA*	serine protease; V8 protease; glutamyl endopeptidase	V8 protease - Exoenzyme	*Staphylococcus aureus* subsp. *aureus* MW2
5	VFG001290	*clfB*	Clumping factor B, adhesin	Clumping factor - Adherence	*Staphylococcus aureus* subsp. *aureus* MW2
6	VFG001281	*sdrE*	Ser-Asp rich fibrinogen-binding bone sialoprotein-binding protein	SDr - Adherence	*Staphylococcus aureus* subsp. *aureus* MW2
7	VFG001279	*sdrC*	Ser-Asp rich fibrinogen-binding bone sialoprotein-binding protein	SDr - Adherence	*Staphylococcus aureus* subsp. *aureus* MW2
8	VFG001316	*geh*	glycerol ester hydrolase	Lipase - Exoenzyme	*Staphylococcus aureus* subsp. *aureus* MW2
9	VFG000077	*clpP*	ATP-dependent Clp protease proteolytic subunit	ClpP - Stress survival	*Listeria monocytogenes* EGD-e
10	VFG001280	*sdrD*	Ser-Asp rich fibrinogen-binding bone sialoprotein-binding protein	SDr - Adherence	*Staphylococcus aureus* subsp. *aureus* MW2
11	VFG046465	*tufA*	elongation factor Tu	EF-Tu - Adherence	*Francisella tularensis* subsp. *tularensis* SCHU S4
12	VFG001289	*clfA*	Clumping factor A, fibrinogen-binding protein	Clumping factor - Adherence	*Staphylococcus aureus* subsp. *aureus* MW2
13	VFG049753	*esaG*	TIGR01741 family protein	Type VII secretion system - Effector delivery system	*Staphylococcus aureus* subsp. *aureus* MW2
14	VFG001292	*hld*	delta-hemolysin	Delta-hemolysin - Exotoxin	*Staphylococcus aureus* subsp. *aureus* MW2
15	VFG049739	*esaG*	TIGR01741 family protein	Type VII secretion system - Effector delivery system	*Staphylococcus aureus* subsp. *aureus* MW2
16	VFG012095	*groEL*	chaperonin GroEL	GroEL - Adherence	*Clostridium difficile* 630
17	VFG049820	*esaG*	TIGR01741 family protein	Type VII secretion system - Effector delivery system	*Staphylococcus aureus* subsp. *aureus* MW2
18	VFG001855	*htpB*	Hsp60, 60K heat shock protein HtpB	Hsp60 - Adherence	*Legionella pneumophila* subsp. *pneumophila* str. Philadelphia 1
19	VFG049780	*esaG*	TIGR01741 family protein	Type VII secretion system - Effector delivery system	*Staphylococcus aureus* subsp. *aureus* MW2
20	VFG049767	*esaG*	TIGR01741 family protein	Type VII secretion system - Effector delivery system	*Staphylococcus aureus* subsp. *aureus* MW2
21	VFG001278	*ebp*	cell surface elastin binding protein	EbpS - Adherence	*Staphylococcus aureus* subsp. *aureus* MW2
22	VFG049805	*esaG*	TIGR01741 family protein	Type VII secretion system - Effector delivery system	*Staphylococcus aureus* subsp. *aureus* MW2
23	VFG001312	*cap8P*	type 8 capsular polysaccharide synthesis protein Cap8P	Capsule - Immune modulation	*Staphylococcus aureus* subsp. *aureus* MW2
24	VFG002189	*cpsB/cdsA*	phosphatidate cytidylyltransferase	Capsule - Immune modulation	*Enterococcus faecalis* V583
25	VFG049826	*esaG*	TIGR01741 family protein	Type VII secretion system - Effector delivery system	*Staphylococcus aureus* subsp. *aureus* MW2
26	VFG048830	*gndA*	NADP-dependent phosphogluconate dehydrogenase	Capsule - Immune modulation	*Klebsiella pneumoniae* subsp. *pneumoniae* NTUH-K2044
27	VFG051098	*clpV/tssH*	type VI secretion system ATPase TssH	T6SS - Effector delivery system	*Acinetobacter baumannii* ACICU

28	VFG049814	*esaG*	TIGR01741 family protein	Type VII secretion system - Effector delivery system	*Staphylococcus aureus* subsp. *aureus* MW2
29	VFG000080	*clpE*	ATP-dependent protease	ClpE - Stress survival	*Listeria monocytogenes* EGD-e
30	VFG049793	*esaG*	TIGR01741 family protein	Type VII secretion system - Effector delivery system	*Staphylococcus aureus* subsp. *aureus* MW2
31	VFG049727	*esaD*	type VII secretion system secreted protein, a nuclease toxin EsaD	Type VII secretion system - Effector delivery system	*Staphylococcus aureus* subsp. *aureus* MW2
32	VFG001281	*sdrE*	Ser-Asp rich fibrinogen-binding bone sialoprotein-binding protein	SDr - Adherence	*Staphylococcus aureus* subsp. *aureus* MW2
33	VFG001280	*sdrD*	Ser-Asp rich fibrinogen-binding bone sialoprotein-binding protein	SDr - Adherence	*Staphylococcus aureus* subsp. *aureus* MW2
34	VFG001279	*sdrC*	Ser-Asp rich fibrinogen-binding bone sialoprotein-binding protein	SDr - Adherence	*Staphylococcus aureus* subsp. *aureus* MW2
35	VFG000079	*clpC*	endopeptidase Clp ATP-binding chain C	ClpC - Stress survival	*Listeria monocytogenes* EGD-e
36	VFG051098	*clpV/tssH*	type VI secretion system ATPase TssH	T6SS - Effector delivery system	*Acinetobacter baumannii* ACICU

Supplementary Table S3. Toxic metabolite-associated genes.

Genomic Location	Orientation	Name	Symbol	Locus	Proteins	Lenth (aa)	Function
Bacillus subtilis MBF10-19J							
NZ_JAGMTL010000001.1:868146-868412	plus	hemolysin XhlA family protein	-	J9345_RS04580	WP_088110622.1	88	Toxin production
NZ_JAGMTL010000001.1:1208543-1209832	plus	hemolysin family protein	*yugS*	J9345_RS06295	WP_041054300.1	429	Toxin production
NZ_JAGMTL010000001.1:1800372-1801700	minus	hemolysin family protein	*yqhB*	J9345_RS09370	WP_017696188.1	442	Toxin production
NZ_JAGMTL010000001.1:2071573-2072214	minus	hemolysin III family protein	*yplQ*	J9345_RS10945	WP_004398522.1	213	Toxin production
NZ_JAGMTL010000001.1:2906560-2906838	minus	hemolysin XhlA family protein	*xhlA*	J9345_RS15535	WP_041344794.1	92	Toxin production
NZ_JAGMTL010000001.1:3189270-3190655	minus	hemolysin family protein	*yhdT*	J9345_RS17075	WP_144452928.1	461	Toxin production
NZ_JAGMTL010000001.1:3192811-3194145	plus	hemolysin family protein	*yhdP*	J9345_RS17090	WP_009966903.1	444	Toxin production
NZ_JAGMTL010000001.1:3551814-3552083	minus	hemolysin XhlA family protein	-	J9345_RS18895	WP_074794633.1	89	Toxin production
NZ_JAGMTL010000001.1:3464924-3468061	minus	surfactin resistance protein SrfP	*srfP*	J9345_RS18505	WP_210216846.1	1045	Lipopeptide production
NZ_JAGMTL010000001.1:3835596-3836324	minus	surfactin biosynthesis thioesterase SrfAD	*srfAD*	J9345_RS20545	WP_017696135.1	242	Lipopeptide production
NZ_JAGMTL010000001.1:3836341-3840168	minus	surfactin non-ribosomal peptide synthetase SrfAC	*srfAC*	J9345_RS20550	WP_210216737.1	1275	Lipopeptide production
NZ_JAGMTL010000001.1:472653-473402	plus	oxygen-insensitive NADPH nitroreductase	*nfsA*	J9345_RS02535	WP_017696211.1	249	Nitrocompound
NZ_JAGMTL010000001.1:2230621-2231229	minus	nitroreductase family protein	*noxC*	J9345_RS12025	WP_014477092.1	202	Nitrocompound
NZ_JAGMTL010000001.1:3305434-3306018	minus	nitroreductase	*yfhC*	J9345_RS17745	WP_210216602.1	194	Nitrocompound
NZ_JAGMTL010000001.1:3629594-3630223	plus	nitroreductase family protein	*ydgI*	J9345_RS19400	WP_041339654.1	209	Nitrocompound
NZ_JAGMTL010000001.1:3648493-3649113	minus	nitroreductase family protein	*ydfN*	J9345_RS19510	WP_015382882.1	206	Nitrocompound
NZ_JAGMTL010000001.1:3805490-3806239	plus	oxygen-insensitive NADPH nitroreductase	*nfsA*	J9345_RS20395	WP_014475811.1	249	Nitrocompound
NZ_JAGMTL010000001.1:3875936-3878251	plus	assimilatory nitrate reductase electron transfer subunit NasB	*nasB*	J9345_RS20650	WP_017696117.1	771	Nitrocompound
NZ_JAGMTL010000001.1:3878258-3880390	plus	assimilatory nitrate reductase catalytic subunit	*nasC*	J9345_RS20655	WP_017696117.1	710	Nitrocompound

Micrococcus luteus MBF05-19J							
NZ_JAGMUB010000001.1:155471-156520	minus	agmatine deiminase family protein	-	J9246_RS00735	WP_135019409.1	349	Biogenic amines production
NZ_JAGMUB010000001.1:924856-926181	plus	hemolysin family protein	-	J9246_RS04280	WP_041103740.1	441	Toxin production
NZ_JAGMUB010000001.1:1417842-1418900	minus	hemolysin family protein	-	J9246_RS06590	WP_041104317.1	352	Toxin production
NZ_JAGMUB010000001.1:1418897-1420213	minus	hemolysin family protein	-	J9246_RS06595	WP_041104315.1	438	Toxin production
NZ_JAGMUB010000001.1:1713434-1714153	plus	hemolysin III family protein	-	J9246_RS08000	WP_069941637.1	239	Toxin production
Staphylococcus hominis MBF12-19J							Toxin production
NZ_JAGMUC010000001.1:932921-933604	plus	hemolysin III family protein	-	J9248_RS04335	WP_017175367.1	227	Toxin production
NZ_JAGMUC010000001.1:2094034-2095404	minus	hemolysin family protein	-	J9248_RS10090	WP_048680802.1	456	Toxin production
NZ_JAGMUC010000001.1:272188-272808	plus	nitroreductase family protein	-	J9248_RS01315	WP_017174732.1	206	Nitrocompound
NZ_JAGMUC010000001.1:719376-723059	plus	nitrate reductase subunit alpha	-	J9248_RS03275	WP_017174959.1	1227	Nitrocompound
NZ_JAGMUC010000001.1:723049-724608	plus	nitrate reductase subunit beta	*narH*	J9248_RS03280	WP_002488804.1	519	Nitrocompound
NZ_JAGMUC010000001.1:724586-725176	plus	nitrate reductase molybdenum cofactor assembly chaperone	*narJ*	J9248_RS03285	WP_093514755.1	196	Nitrocompound
NZ_JAGMUC010000001.1:725169-725858	plus	respiratory nitrate reductase subunit gamma	*narI*	J9248_RS03290	WP_017174958.1	229	Nitrocompound
Staphylococcus warneri MBF02-19J							
NZ_JAGMUP010000001.1:1099160-1099843	plus	hemolysin III family protein	-	J9233_RS05280	WP_210225249.1	227	Toxin production
NZ_JAGMUP010000001.1:2391075-2392424	minus	hemolysin family protein	-	J9233_RS11670	WP_002467170.1	449	Toxin production
NZ_JAGMUP010000012.1:59617-60633	plus	hemolysin family protein	-	J9233_RS12240	WP_041786899.1	338	Toxin production
NZ_JAGMUP010000001.1:888689-889318	plus	nitroreductase family protein	-	J9233_RS04150	WP_002467276.1	209	Nitrocompound
NZ_JAGMUP010000001.1:1232422-1233048	minus	nitroreductase family protein	-	J9233_RS05980	WP_002466261.1	208	Nitrocompound
NZ_JAGMUP010000001.1:2281931-2282470	minus	nitroreductase	-	J9233_RS11120	WP_002465937.1	179	Nitrocompound
NZ_JAGMUP010000001.1:877070-880753	plus	nitrate reductase subunit alpha	-	J9233_RS04100	WP_210225220.1	1227	Nitrocompound
NZ_JAGMUP010000001.1:880743-882296	plus	nitrate reductase subunit beta	*narH*	J9233_RS04105	WP_015364729.1	519	Nitrocompound
NZ_JAGMUP010000001.1:882274-882864	plus	nitrate reductase molybdenum cofactor assembly chaperone	*narJ*	J9233_RS04110	WP_095324974.1	196	Nitrocompound
NZ_JAGMUP010000001.1:882857-883534	plus	respiratory nitrate reductase subunit gamma	*narI*	J9233_RS04115	WP_002465461.1	229	Nitrocompound

Supplementary Table S4. *In vitro* antibiotic resistance profiles and antimicrobial activity of the four sequenced bacterial genomes.

Strain	*In Silico*	*In Vitro*
***Bacillus subtilis* MBF10-19J**	**Resistant** to<br>● **Vancomycin** (CARD: *vanY* in *vanM* cluster, *vanW* in *vanI* cluster, *vanT* in *vanG* cluster)<br>● **Macrolides** (CARD: *tmrB*, *mphK*; Resfinder: *mphK*)<br>● **Chloramphenicol** (CARD: *ykkD* and *ykkC*)<br>● **Amoxicillin** (ResFinder: *blaZ*)<br><br>**Positive** for subtilosin A and SboX bacteriocin loci	● **Resistant** to **vancomycin** (MIC = 32 μg/mL), but inhibition zones indicates susceptibility (⌀25,15 mm). There is **no breakpoint guideline** (EUCAST or CLSI) **vancomycin** for ***Bacillus subtilis***.<br>● **Sensitive** to **macrolides** (disc diffusion gentamycin ⌀27,58 mm, erythromycin ⌀24,32 mm)<br>● **Sensitive** to **chloramphenicol** (disc diffusion: ⌀35,35 mm)<br>● **Resistant** to **amoxicillin** (MIC = 50 μg/mL, disc diffusion: ⌀8,05 mm)<br>Positive antimicrobial activity (in mm)<br>● *P. aeruginosa*: 2.32 ∣ 2.47

		• *E. coli*: 2.37 ǀ 2.46<br>• *S. mutans*: 2.22 ǀ 2.25<br>• *S. aureus*: 2.37 ǀ 2.45<br>• *S. typhimurium*: 2.20 ǀ 2.30<br>• *B. subtilis*: 2.15 ǀ 2.10
Micrococcus luteus **MBF05-19J**	**Resistant** to **Vancomycin** (*vanY* in *vanA* cluster)<br><br><br>**Negative** for bacteriocin genes	**Vancomycin intermediate** susceptibility (MIC = 8 μg/mL), but **zone inhibition diameter** shows **sensitivity** to vancomycin (ø26,82 mm). There is **no official breakpoint guideline** vancomycin for *Micrococcus luteus*.<br>Positive antimicrobial activity (in mm)<br>• *P. aeruginosa*: 2.07 ǀ 2.17<br>• *E. coli*: 2.17 ǀ 2.12<br>• *S. mutans*: 2.07 ǀ 2.05<br>• *S. aureus*: 2.17 ǀ 2.23<br>• *S. typhimurium*: 2.07 ǀ 2.15<br>• *B. subtilis*: 1.90 ǀ 1.88
Staphylococcus hominis **MBF12-19J**	**Resistant** to<br>• **vancomycin** (CARD: *vanY* in *vanG* cluster, *vanT* in *vanG* cluster)<br>• **fluoroquinolone** (CARD & ResFinder: *fusC*)<br>• **amoxicillin** (ResFinder: *blaZ*)<br>Positive for auto inducing peptide I	• **Sensitive** to **vancomycin** (MIC 4 μg/mL; ø31,10 mm)<br>• **Sensitive** to **fluoroquinolones** (disc diffusion: ø44,05 mm)<br>• **Sensitive** to **amoxicillin** (disc diffusion: ø37,40 mm)<br><br>Positive antimicrobial activity (in mm)<br>• *P. aeruginosa*: 2.37 ǀ 2.37<br>• *E. coli*: 2.27 ǀ 2.23<br>• *S. mutans*: 2.22 ǀ 2.25<br>• *S. aureus*: 2.37 ǀ 2.27<br>• *S. typhimurium*: 2.47 ǀ 2.35<br>• *B. subtilis*: 2.27 ǀ 2.24
Staphylococcus warneri **MBF02-19J**	**Resistant** to<br>• **vancomycin** (CARD: *vanT* in *vanG* cluster)<br>• **fluoroquinolone** (CARD: *sdrM*)<br><br>**Positive** for warnericin (bacteriocin), autoinducing peptide II, and delta lysin	• **Intermediate** susceptibility to **vancomycin** (MIC 8 μg/mL). Disc diffusion shows sensitivity to vancomycin (ø24,27 mm)<br>• **Sensitive** to **fluoroquinolones** (ø39,48 mm)<br>Positive antimicrobial activity (in mm)<br>• *P. aeruginosa:* 2.15 ǀ 2.17<br>• *E. coli:* 2.20 ǀ 2.12<br>• *S. mutans:* 2.25 ǀ 2.12<br>• *S. aureus:* 2.30 ǀ 2.27<br>• *S. typhimurium:* 2.07 ǀ 2.17<br>• *B. subtilis:* 2.02 ǀ 2.00
